# Common Predictive Factors of Social Media Addiction and Eating Disorder Symptoms in Female College Students: State Anxiety and the Mediating Role of Cognitive Flexibility/Sustained Attention

**DOI:** 10.3389/fpsyg.2021.647126

**Published:** 2022-03-29

**Authors:** Zhonghua He, Mingde Li, Chanjun Liu, Xiaoyue Ma

**Affiliations:** School of Journalism and New Media, Xi’an Jiaotong University, Xi’an, China

**Keywords:** social media addiction, female college students, state anxiety, cognitive flexibility, sustained attention, common predictive factors, eating disorder symptoms

## Abstract

This study aimed to investigate the common predictive factors between social media addiction (SMA) and eating disorder symptoms (EDS), in a group of Chinese female college students. A total of 216 students completed the behavioral assessments of cognitive flexibility and sustained attention, as well as the questionnaires on anxiety, social media dependence, and eating disorders. The results indicate that SMA is significantly correlated with EDS. Structural equation modeling was used to test the model in which state anxiety, cognitive flexibility, and sustained attention predicted social gain and EDS. Additionally, the results confirmed the mediating role of cognitive flexibility and sustained attention between state anxiety and SMA/EDS in the participants. The findings revealed that in the sample group, state anxiety was related to SMA and EDS through cognitive flexibility and sustained attention. These proposals reflect the significance of improving cognitive flexibility/sustained attention and reducing state anxiety to prevent EDS and SMA in female college students.

## Introduction

In modern society, social media has become an indispensable part of the daily lives of people. Compulsive social media use has repercussions on the social, psychological, professional, and personal lives of the users (Abbasi, 2018). Its widespread use has increased the ease of interpersonal communication between individuals and socialization processes; yet, it has also led to social media addiction (SMA) emerging as a problem (Kumpasolu et al., 2021). When individuals are so engaged in social media that they feel distressed when they are unable to use it, such misuse is widely referred to as SMA (Hou et al., 2019; Hussain and Starcevic, 2020). Most social media users constitute young adults at the age of 18–24 years (Ramesh-Masthi et al., 2018). SMA is particularly prominent in college students, due to early contact and widespread usage (Zendle and Bowden-Jones, 2019).

Online social media behaviors (e.g., viewing and commenting) have been significantly correlated with a drive for thinness (DT) among female undergraduate students (Ji and Chock, 2015). Eating disorder symptoms (EDS), especially among young adults, have become a worldwide concern (Alpaslan et al., 2015). Many social, cultural, and psychological factors are associated with eating attitudes and behaviors. Social media sites, such as Weibo, are among the most widespread and used online communication networks (Navithasulthana and Shanmugam, 2021). Previous studies found that people who use Facebook more were less satisfied with their appearance (Stronge et al., 2015) and reported more eating disorders (Mabe et al., 2014). Conversely, women with EDS also come in contact with social media and continue perpetuating the disorder, believing that EDS are a lifestyle rather than a mental health condition (Eikey, 2017). Positive correlations have also been observed between social networking site (SNS) usage and disordered eating levels in young adults (Mabe et al., 2014; Hummel and Smith, 2015). In developing countries, mass media exposure has a significant impact on eating attitudes and behaviors (Eapen et al., 2006; Musaiger et al., 2013).

Interestingly, SMA has been associated with decreased life satisfaction and increased anxiety in young adults (O’Day and Heimberg, 2021). Social media and food cues have been shown to activate the same brain regions, such as the orbitofrontal cortex, nucleus accumbens, the amygdala, anterior cingulate, striatum, and medial frontal cortex (Dagher, 2009; Gearhardt et al., 2011). Given the rapid growth of SNS usage and the potential associations between SMA and EDS, research on common predictive factors of the two is needed.

Among all the factors, anxiety is often inextricably linked with and SMA/EDS. Because the symptoms of anxiety may precede EDS, predict poor outcome, and persist even after an individual has recovered from the disorder, it is important to identify factors that, in interaction with anxiety, are associated with eating disorders (Fitzsimmons and Bardone-Cone, 2011). People with high anxiety levels reported a preference for online interaction. For instance, socially anxious individuals, who were briefly able to chat with an individual online before meeting them in person, experienced reduced anxiety (Caplan, 2007; Markovitzky et al., 2012). This may especially hold true for those who use disordered eating behaviors and social media as a response to anxiety. Accordingly, points of intervention should focus on relieving anxiety or on identifying whether certain factors associated with anxiety could lessen its effect on poor outcomes (e.g., addictive SNS usage and disordered eating) and examining potential mediating factors of the relationship between anxiety and those negative consequences.

The ability to concentrate on, choose, and accomplish a primary target is largely based on a series of cognitive functions, termed executive functions (EFs; Sheridan et al., 2012). Overall, EFs involve flexible switching between strategies, the inhibition of interference disturbance, organizing, and planning strategies (Robbins, 1996). The importance of cognitive flexibility as a fundamental aspect of health, as well as in the field of EDs (Smith et al., 2018), has gained widespread emphasis in recent years (Rutten et al., 2013). As with food and drug addiction (Albein-Urios et al., 2012; Christopher et al., 2018), EF likely plays key roles in SMA and EDS. For instance, subjects with internet addiction disorder engage more in attention shifting, as well as show impaired cognitive flexibilities, than healthy controls (Dong et al., 2014). Significant differences exist in the flexibility of people with and without EDs (Dahlgren et al., 2019).

Schmeichel and Baumeister (2010) defined sustained attention as “focusing attention on a stimulus or activity for an extended period of time” (Schmeichel and Baumeister, 2010). Sustained attention has been found to be one of the major variables inducing behavioral problems (Barker et al., 2009). Multiple studies have shown that inattention fosters binge eating (Cortese et al., 2007; Reinblatt, 2015). In such cases, insufficient sustained attention might result in continual social media use, especially when there are push notifications or voice reminders (Witkiewitz and Villarroel, 2009).

Studies have also shown that a negative affective state greatly influences and alters EFs, which is reflected in different tasks such as the planning test, sustained attention tasks, and cognitive flexibility tasks (Holmes and Pizzagalli, 2007; He and Yin, 2016). Anxiety has also been shown to affect EF performance in other psychiatric populations (Wu et al., 2011; Demetriou et al., 2020). For EF, women who performed poorly were likely to have reported higher levels of state anxiety (Billingsley-Marshall et al., 2013). In addition, one report suggests that at least one type of EF, i.e., cognitive flexible switching, is diminished in anxious women with EDS (Roberts et al., 2010).

Internet use is the highest among adolescents and young adults (Alpaslan et al., 2015). Young adults primarily use social media for communication, entertainment, and professional development, making it indispensable for university students (Mohammadbeigi et al., 2011). Life habits are usually formed during young adulthood (Çelik et al., 2015). College students are, thus, at risk of disordered eating attitudes due to the elevated mental and physical demands of higher education (Rianna et al., 2021).

Therefore, disordered eating is a significant problem in college individuals, especially female undergraduate students. EDS affect 13% of females and contribute to functional impairment and mortality (Eric et al., 2020). Young women increasingly spend considerable time on social media (Eckler et al., 2016). More time spent on Facebook relates to the more frequent body and weight comparisons, more attention to the physical appearances of others, and more negative feelings about their own bodies (Kim and Park, 2016). For women who want to lose weight, more time on social media relates to more disordered eating symptoms (Eckler et al., 2016). Additionally, previous studies showed a positive relationship between experiencing negative emotions and eating disorder psychopathology in women (Magel and Ranson, 2021).

Social media has changed and challenged the way we interact with each other (Hilsen and Helvik, 2014). Because social gain (SG) was considered an outcome, which would be the most likely potential pathway from social media use to EDS risk, we chose SG of SMA (SMA-SG) as the main object for research. In this study, SMA-SG refers to the improvement of interpersonal relationships, which college students experience through mobile social media use. Considering the correlations between the performance of emotionally interfered EF and risk behaviors associated with health (Galindo et al., 2019; Lozano-Madrid et al., 2020) and the impact of anxiety on EFs (Demetriou et al., 2020; Warren et al., 2021), we speculated on the mediating roles of cognitive flexibility and sustained attention among anxiety, SMA-SG, and EDS outcomes.

**FIGURE 1:**
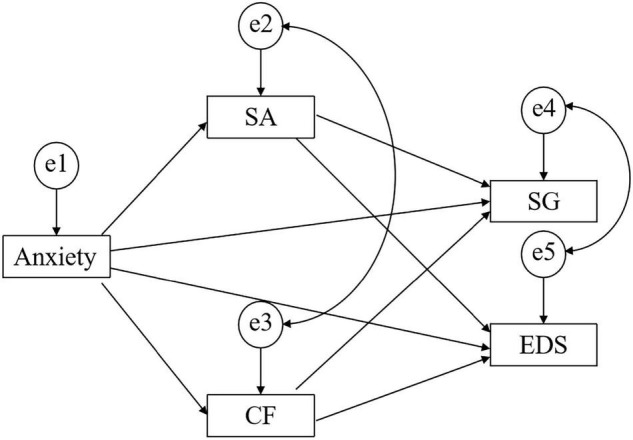
A theoretical model relating state anxiety (Anxiety) to social gain (SG) and eating disorder symptoms (EDS). CF, Cognitive Flexibility; SA, Sustained Attention.

Based on our proposed mediation model (Figure 1), we expected to find: (a) direct associations between both state anxiety, and SMA-SG and EDS; (b) direct associations between state anxiety and sustained attention/cognitive flexibility; (c) direct associations between sustained attention, cognitive flexibility and SMA-SG, and EDS; and (d) indirect, mediated paths from state anxiety through cognitive flexibility/sustained attention to SMA-SG and EDS. Because we considered sustained attention and cognitive flexibility to be two processes linking anxiety with SMA-SG and EDS, we hypothesized that partial mediation would be present, indicating that the effects of state anxiety on SMA-SG and EDS remain significant in a model that accounts for mediation by sustained attention and cognitive flexibility.

## Materials and Methods

### Participants

The sample consisted of 216 female undergraduate students including sophomores (21.06%), juniors (49.12%), and seniors (29.82%) (mean age = 19.8 years; age range = 19–22 years) who signed informed consent forms. The mean body mass index (BMI) (kg/m^2^) was 20.78 and ranged from 17.00 to 25.04. A total of 216 Chinese female undergraduates were recruited from Xi’an of Shaanxi province through advertisements at local universities. All procedures performed in studies involving human participants were in accordance with the ethical standards of the Biomedical Ethics Committee of the Medical Department of Xi’an Jiaotong University. Inclusion criteria were those with no reported significant medical impairments or physical/mental problems. These were verified *via* questionnaire items, stating that the female undergraduates have never been diagnosed with eating disorders at the time of joining the study, nor have they undergone pharmacological treatment and/or participated in individual or group psychotherapy due to mental problems (e.g., learning disabilities, diagnosed eating disorders, and anxiety disorders), and are not visibly handicapped or physically deformed. All the female undergraduates met the inclusion criteria and completed the behavioral experiments and questionnaires (detailed below). Every participant received monetary compensation (¥ 80) for their time and effort on the completion of the tasks.

### Procedure

The participants individually accepted tests in a quiet room. They completed tasks in the following fixed order: the Trail Making Test (TMT), Letter Cancel Test (LCT), State-Trait Anxiety Inventory (STAI), the Social Media Dependence Questionnaire—Social Gain subscale, and the Eating Disorder Inventory. Each measure is described in detail in the section “Measures.” The complete procedure took about 15–25 min.

### Measures

#### Cognitive Flexibility

The TMT was used to measure the cognitive flexibility of participants (Sarsour et al., 2011) and was administered in two parts. TMT Part A is a visual-scanning task in which the participant was required to quickly draw lines sequentially connecting consecutive numbered circles (1–25 circles randomly arranged on a page). TMT Part B added a measure of cognitive flexibility by asking the participant to connect the same number of circles in an alternating sequence of numbers and letters (e.g., 1, A, 2, B). Both parts of the tests were timed. For this analysis, we used a difference score defined as △TMT [(Part B)-(Part A)]. The △TMT score is used to control the effect of motor speed on TMT performance and is considered a more accurate measure of cognitive flexibility than Part B (Corrigan and Hinkeldey, 1987; Lezak, 1995). Lower scores indicated better performance.

#### Sustained Attention

The LCT was used to evaluate the ability of participants to provide continuous attention. A randomly distributed alphabet contains 22 × 39 letters. Stipulates “a” and “M” are the Cancel Targets; there are a total of 97 Cancel Targets. The participant was required to check the alphabet line by line and cancel the target letter as quickly and accurately as possible. The longest completion time did not exceed 7 min. The calculation formula for the Cancel Test was as follows: *Q* score = (number of correct responses/numbers of total Cancel Targets) × (correct number of reactions/viewing time) (Kimberga and D’Esposito, 2003).

#### State Anxiety

The levels of state anxiety were measured with the Chinese version of the STAI State Anxiety Scale (S-Anxiety) (Shek, 1988; Julian, 2011). There are two subscales within this measure. The S-Anxiety evaluates the current state of anxiety, asking how respondents feel “right now,” using items that measure the subjective feelings of apprehension, tension, nervousness, worry, and activation/arousal of the autonomic nervous system. The S-Anxiety has 20 items that are self-rated on a 4-point Likert scale, ranging from 1 (not at all) to 4 (very much); for each respondent, these are added to obtain a total score, which ranges from 20 to 40, with higher scores indicating higher levels of state anxiety. The scale has been tested and validated for the Chinese context. The S-Anxiety exhibited satisfactory psychometric qualities, and Cronbach’s alpha was 0.85.

#### Social Gain of Social Media Addiction

The Mobile Social Media Dependence Questionnaire measures five different dimensions using five subscales, namely, SG, salience, compulsivity, conflict, and withdrawal. This SG variable was measured through 5 items (Wu, 2014) as follows: “Communicating with others using mobile social media leads to better confidence and is easier for me than in reality,” “Mobile social media meet most of my social needs,” “Mobile social media gives me more attention and influence than I receive in reality,” “The attention and comments of others on mobile social media make me feel very fulfilled,” and “When I am lonely, I like to communicate with others on mobile social media.” All five items are answered on a 5-point Likert scale ranging from strongly disagree (1) to totally agree (5). Total scores on the SG ranged from 5 to 25, with higher scores denoting higher levels of SG. This questionnaire exhibited satisfactory psychometric qualities, and Cronbach’s alpha was 0.77.

#### Eating Disorder Symptoms

The Eating Disorder Inventory-1 (EDI-1) was compiled by Garner (1983) and has good reliability and validity in China (Lee et al., 1998; Hui et al., 2019). It includes eight subscales, namely, DT, Bulimia (B), Body Dissatisfaction (BD), Ineffectiveness (I), Perfection (P), Interpersonal Distrust (ID), Interoceptive Awareness (IA), and Maturity Fears (MF). The EDI-1 has 64 items, self-rated on a 6-point Likert scale (1 = always, 2 = usual, 3 = often, 4 = sometimes, 5 = very less, and 6 = never). The lower the subscales score, the more serious the related problem is. In this study, the EDI and all subscales presented with adequate levels of reliability (EDS α = 0.85; DT α = 0.81; B α = 0.70; BD α = 0.73; I α = 0.83; P α = 0.75; ID α = 0.71; IA α = 0.71; and MF α = 0.77).

### Data Analyses

The means, SDs, correlations, and path analyses were computed using SPSS 16.0 and AMOS 7.0 software (Arbuckle, 2006). Mediation tests indicated whether the association between two or more variables resulted from another variable or a set of variables. Path analysis is a specific tool of the structural equation modeling (SEM) analysis to analyze the assumed relationships of multivariate data. In this study, we employed a strictly confirmatory strategy using SEM. The hypothesized model was tested using the path analysis procedure with the aid of AMOS 7.0 (Arbuckle, 2006). The following goodness-of-fit statistics were analyzed. The normed chi-square test is used to compare the magnitude of the chi-square value with the degrees of freedom (χ^2^/df). For a good fit, this proportion should be as small as possible; values less than 3 indicate a good or acceptable fit (Schermelleh-Engel et al., 2003). An increase in the root mean square error of approximation (RMSEA) exceeding 0.01 suggests non-invariance and leads us to reject the model (Cheung and Rensvold, 2002; Chen, 2007). Incremental fit indices measure the improvement of fit by comparing the proposed model with one that assumes no association between the observed variables, referred to as the independence model. The incremental fit indices include the normed fit index (NFI), the comparative fit index (CFI), and the incremental fit index (IFI), and the values of these indices should be close to 1.0 to indicate a good fit.

## Results

### Descriptive Statistics

Means and SDs for the study variables and their correlations are presented in Table 1. Means and SDs for the EDS and subscales of EDI-1 were significantly lower than other age-matched clinical samples and close to the scores of other non-clinical ones (Zhang and Kong, 2004). Regarding social media dependence level, mild dependence (9.55–13.05) accounted for 38.89% of the participants (*n* = 84) and moderate dependence (13.25–16.75) accounted for 37.04% of the participants (*n* = 80) (Wu, 2014); therefore, three quarters met the level of mild-to-moderate dependence. Table 1 provides the correlations included in the hypothesized model. As can be seen from this table, the majority of expected correlations were found (Table 1).

**TABLE 1:** Partial correlations for anxiety, cognitive flexibility, sustained attention, social gain, and eating disorder symptoms after controlling for age.

	Mean (SD)	1	2	3	4	5	6	7	8	9	10	11	
1. Anxiety	38.33 (8.53)												
2. CF(s)	54.52 (45.86)	0.23**											
3. SA	4.87 (0.97)	−0.13^†^	−0.18**										
4. SG	12.45 (3.05)	0.24**	–0.03	−0.18**									
5. DT	27.46 (7.09)	−0.18**	−0.21**	0.04	−0.19**								
6. B	34.65 (5.37)	–0.05	0.06	–0.01	−0.37**	0.11							
7. BD	26.79 (10.48)	−0.22**	−0.14*	0.09	–0.07	0.61**	0.18**						
8. I	40.56 (7.31)	−0.62**	−0.18**	0.16*	−0.13^†^	0.27**	0.19**	0.40**					
9. P	19.53 (4.43)	−0.23**	0.08	–0.04	−0.31**	0.32**	0.38**	0.23**	0.01				
10. ID	28.98 (5.67)	−0.36**	−0.14*	0.15*	–0.02	0.06	0.17*	0.12^†^	0.68**	–0.06			
11. IA	44.12 (7.15)	−0.49**	–0.09	0.05	−0.26**	0.36**	0.40**	0.36**	0.62**	0.42**	0.38**		
12. MF	26.98 (7.22)	–0.10	0.07	0.20**	−0.34**	0.29**	0.37**	0.26**	0.12^†^	0.37**	0.06	0.29**	
13. EDS	249.06 (34.08)	−0.46**	−0.13^†^	0.14*	–0.32	0.80**	0.21**	0.89**	0.57**	0.25**	0.42**	0.49**	0.26**

^†^p < 0.10; *p < 0.05, **p < 0.01.

*Anxiety, State Anxiety; CF, Cognitive Flexibility; SA, Sustained Attention; SG, Social Gain; DT, Drive for Thinners; B, Bulimia; BD, Body Dissatisfaction; I, Ineffectiveness; P, Perfection; ID, Interpersonal Distrust; IA, Interoceptive Awareness; MF, Maturity Fears; EDS, Eating Disorder Symptoms.*

### Testing the Model

**FIGURE 2:**
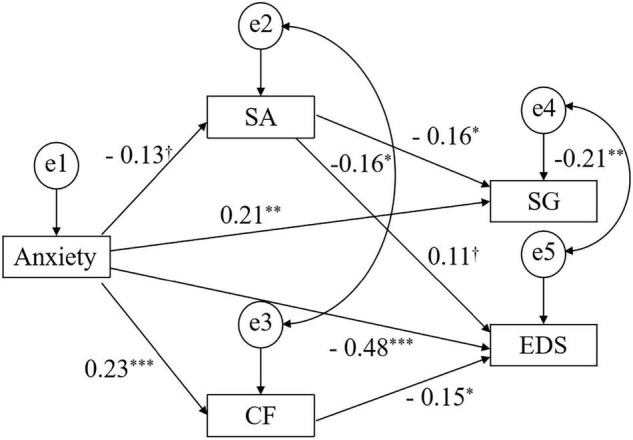
Significant and insignificant paths from state anxiety to SG and EDS (using bias-corrected bootstrapped CIs). Mean path coefficients were obtained using 2,000 bootstrap samples. ^†^*p* < 0.10, *p < 0.05, **p < 0.01, ***p < 0.001. Anxiety, State Anxiety; CF, Cognitive Flexibility; SA, Sustained Attention; SG, Social Gain; EDS, Eating Disorder Symptoms.

According to the criterion of mediating variable analyses, which was described previously (Kraemer et al., 2001), all main variables were included in the path analysis with the exception of the pathways from cognitive flexibility to SG. The path analysis aimed to verify whether the relationships between state anxiety and EDS/SG were mediated by their cognitive flexibility and sustained attention. Considering the correlation results among different variables and the sample size in this study, observed variables were eventually included in the hypothesized model. The indices suggested that the model had a reasonable fit to the data: χ^2^/df = 2.81, RMSEA = 0.092, CFI = 0.98, IFI = 0.98, and NFI = 0.97. Figure 2 presents the test results of the hypothesized model. The exact significance values of each pathway as well as the SEs and standardized coefficients are shown in Table 2.

**TABLE 2:** Coefficients, SEs, and significance values of each pathway.

	Coefficient	SE	*p*
Anxiety → SA	–0.13	0.0077	0.0631
Anxiety → CF	0.23	0.3571	<0.001
Anxiety → SG	0.21	0.0047	0.0011
Anxiety → EDS	–0.48	0.2441	<0.001
SA → SG	–0.16	0.0415	0.0181
SA → EDS	0.11	2.1165	0.0714
CF → EDS	–0.15	0.0451	0.0117
e2 ↔ e3	–0.16	2.9671	0.0198
e4↔ e5	–0.21	1.2181	0.0022

*Anxiety, State Anxiety; CF, Cognitive Flexibility; SA, Sustained Attention; SG, Social Gain; EDS, Eating Disorder Symptoms.*

## Discussion

In this study, we examined the relationship between state anxiety and the EDS/SMA-SG, as well as the mediating effect of EFs (i.e., cognitive flexibility and sustained attention) between state anxiety and EDS/SMA-SG.

We found that high-anxiety female undergraduates were significantly more addicted to social media and prone to EDS than those with low anxiety. Therefore, state anxiety is a common predictive factor of SMA and EDS. These results are partially consistent with previous findings (Fitzsimmons and Bardone-Cone, 2011; Ko et al., 2014; Moosavi and Amini, 2017), confirming the role of negative emotion in developing risky behavior and improper habits.

This study verified the previous conclusion of a significant correlation between SMA and EDS (Ji and Chock, 2015; Stronge et al., 2015). Most previous studies were cross-sectional and exploratory in nature, thus preventing conclusion about causation (Wilksch et al., 2020). There have been few longitudinal studies, as the longitudinal design does not have the same internal validity as an experimental design (de Vries et al., 2016). This study differs from most earlier ones in that the previous design, such as the two-wave panel study, has not been applied to young adult women. Furthermore, another possible relationship exists that those individuals who develop EDS may consequently use more social media. Therefore, in the research hypothesis and verification process of this study, SMA and EDS can only be used as equally important outcome variables.

Until recently, cognitive flexibility and sustained attention were not considered as possible factors that influence eating attitudes/behaviors and addiction in young women who were high in state anxiety. Studies have confirmed the high risk of eating disorders and addiction in women with deficits in cognitive abilities (Goldstein et al., 2007; Cury et al., 2020). Therefore, we investigated the relationship between state anxiety, cognitive flexibility, sustained attention, and EDS/SMA-SG. SEM is a strong analytical method for examining complex relationships using multivariate data. Furthermore, unlike regression analysis, SEM reveals indirect effects.

The findings regarding the mediating effect of cognitive flexibility and sustained attention in female college students were preliminary but meaningful. In the relationship between state anxiety and EDS/SMA-SG, SEM confirmed the mediating role of cognitive flexibility and sustained attention in young women, thus confirming the relevant hypothesis. The finding that cognitive flexibility is a potential pathway for the impact of state anxiety on EDS and is consistent with previous research, which emphasized the importance of preventing the deterioration of EF deficits in individuals with state anxiety in an inpatient EDS program (Billingsley-Marshall et al., 2013). State anxiety also appears to contribute to diminished EF in women with eating disorders (Billingsley-Marshall et al., 2013). An increase in cognitive flexibility could help patients accept changes in their habits and maintain behaviors, with a possible long-term effect (Dingemanse et al., 2013). The potential of neurocognitive training programs for EDS is high, given the combined evidence of the role of deficits in executive functioning in EDS (i.e., the training of cognitive flexibility not only fosters performance in a test of cognitive flexibility but also seems to impact flexibility in the daily lives of participants) (Brockmeyer et al., 2013; Juarascio et al., 2015), which indicates the possibility of specific neuropsychological training in EDS. Consider that cognitive flexibility showed a significant improvement with the cognitive remediation therapy (CRT) rolling protocol (Meneguzzo et al., 2021). We speculated that cognitive flexibility treatment would have significant effects in the non-clinical population in the future.

Capacity theories propose that a negative affective state will increase cognitive resource load (Seibert and Ellis, 1991). The results of this study indicated that difficulties in sustained attention and cognitive flexibility are associated with increased state anxiety in female college students. This phenomenon reflects that when fulfilling a task requiring mental flexibility and sustained attention, which contributes to avoiding social media-based interruption and being flexible about BD and DT, the increase in the negative affective state could, in fact, affect information processing (Gasper and Clore, 2002).

The findings of this study should be interpreted in light of several limitations. First, this research used cross-sectional data for the analysis, thereby making the direction of causality difficult to determine. Therefore, future studies that employ longitudinal data and an experimental design must confirm the direction of causality. Second, the data about state anxiety and EDS/SMA-SG were self-reported. However, because respondents were assured that their responses were confidential, it is unlikely that respondents were not truthful. Third, because this sample consisted of young college students aged 19–22 years and no clinical samples were included, results cannot be generalized to all the youth and clinical populations. Future research will need to cover a diverse sampling of people for investigation.

In summary, the results of this study supported the idea that state anxiety is a common predictive factor of SMA and EDS. Importantly, these results showed that cognitive flexibility and sustained attention of female college students mediated the association between state anxiety and SMA-SG/DS. Therefore, the obstructive effect of the deficits in cognitive flexibility and sustained attention in the prevention of EDS and SMA should be seriously considered. These proposals reflect the significance of improving affective state and reducing state anxiety to prevent EDS and SMA in female college students.

## Data Availability Statement

The data used during the current study are available from the corresponding author on reasonable request.

## Ethics Statement

The studies involving human participants were reviewed and approved by the ethical standards of Biomedical Ethics Committee of the Medical Department of Xi’an Jiaotong University. The patients/participants provided their written informed consent to participate in this study.

## Author Contributions

ZH and ML identified the research question and designed the study. ZH collected and analysed the data and discussed with all the authors. All authors made significant contributions, read, and approved the final manuscript.

## Conflict of Interest

The authors declare that the research was conducted in the absence of any commercial or financial relationships that could be construed as a potential conflict of interest.

## Publisher’s Note

All claims expressed in this article are solely those of the authors and do not necessarily represent those of their affiliated organizations, or those of the publisher, the editors and the reviewers. Any product that may be evaluated in this article, or claim that may be made by its manufacturer, is not guaranteed or endorsed by the publisher.
